# A partially demineralized allogeneic bone graft: in vitro osteogenic potential and preclinical evaluation in two different intramembranous bone healing models

**DOI:** 10.1038/s41598-021-84039-6

**Published:** 2021-03-01

**Authors:** Pierre Tournier, Jérôme Guicheux, Arnaud Paré, Aymeric Maltezeanu, Thibaut Blondy, Joëlle Veziers, Caroline Vignes, Manon André, Julie Lesoeur, Ana Barbeito, Raphaël Bardonnet, Christophe Blanquart, Pierre Corre, Valérie Geoffroy, Pierre Weiss, Alexis Gaudin

**Affiliations:** 1 INSERM, UMR 1229, RMeS, Regenerative Medicine and Skeleton, Université de Nantes, Oniris, 1 Place Alexis Ricordeau, 44042 Nantes, France; 2 BIOBank SAS, Lieusaint, France; 3 INSERM, UMR 1229, RMeS, Regenerative Medicine and Skeleton, CHU Nantes, Université de Nantes, Oniris, 1 Place Alexis Ricordeau, 44042 Nantes, France; 4 INSERM, UMS 016, CNRS 3556, Structure Fédérative de Recherche François Bonamy, SC3M Facility, CHU Nantes, Université de Nantes, 44042 Nantes, France; 5 Service de Chirurgie Maxillo-faciale, Plastique et Brulés, Hôpital Trousseau, CHU de Tours, 37170 Tours, France; 6 Université de Nantes, Univ Angers, INSERM, CNRS, CRCINA, 44000 Nantes, France

**Keywords:** Cell biology, Immunology, Stem cells, Medical research

## Abstract

In skeletal surgical procedures, bone regeneration in irregular and hard-to-reach areas may present clinical challenges. In order to overcome the limitations of traditional autologous bone grafts and bone substitutes, an extrudable and easy-to-handle innovative partially demineralized allogenic bone graft in the form of a paste has been developed. In this study, the regenerative potential of this paste was assessed and compared to its clinically used precursor form allogenic bone particles. Compared to the particular bone graft, the bone paste allowed better attachment of human mesenchymal stromal cells and their commitment towards the osteoblastic lineage, and it induced a pro-regenerative phenotype of human monocytes/macrophages. The bone paste also supported bone healing in vivo in a guide bone regeneration model and, more interestingly, exhibited a substantial bone-forming ability when implanted in a critical-size defect model in rat calvaria. Thus, these findings indicate that this novel partially demineralized allogeneic bone paste that combines substantial bone healing properties and rapid and ease-of-use may be a promising alternative to allogeneic bone grafts for bone regeneration in several clinical contexts of oral and maxillofacial bone grafting.

## Introduction

In oral and maxillofacial bone grafting procedures, autologous bone grafting is still considered to be the reference technique. This technique has a high success rate as a result of the combination of osteoconductivity (the ability to provide a structural support for bone growth), osteogenicity (the promotion of osteoblastic differentiation of progenitor cells), and osteoinductivity (the induction of bone growth in large bone defects or heterotopic sites, mainly due to growth factors in the bone and marrow environment)^1–4^. Despite these advantages, autologous bone grafts (ABGs) suffer from numerous serious peri- and post-operative drawbacks. Among these, the potential donor site morbidities (e.g., pain, hematoma, blood loss, nerve injury, the risk of bone fracture), the additional operative time, the limited available bone (e.g., iliac crest, calvarial bone), and the occasional need for a second surgical team are the main limitations of this procedure and consequently hindering the clinical benefits. To overcome these limitations, several alternatives such as alloplastic and xenogeneic bone substitutes or allogeneic bone grafts are used in oral and maxillofacial surgeries^5–7^.

Allogeneic bone grafts are reliable and have been used extensively for decades as an alternative to ABGs^8,9^. The amount of allogeneic bone is virtually unlimited, and it can be obtained in various sizes and shapes (e.g., blocks, blades, paste, putty, powder, chips, injectable forms). The duration of the surgery is shortened compared to ABG-based reconstructions because there is no need for a bone harvesting procedure. Moreover, allogeneic bone grafts exhibit the same bone healing capacities as ABGs in multiple indications such as bone augmentation before dental implant placement (e.g., sinus floor elevation, ridge augmentation)^10,11^.

Although allogeneic bone grafts are mainly used in powder or block forms, they exhibit a lack of versatility and/or handling, especially in specific indications related to pre-implant surgery, such as sinus floor elevation, guided bone regeneration in horizontal/vertical augmentation, and alveolar socket preservation. To address these unmet clinical needs, a novel ready-to-use, easy-to-handle, extrudable, and moldable human bone graft for bone regenerative medicine has been developed^12^. This allogeneic bone paste is made of partially demineralized bone particles, consisting of a mineralized core (Sup. Fig. 1, upper panel, white arrows) surrounded by a demineralized bone matrix (Sup. Fig. 1, upper panel, black arrows). This bone paste does not require any mixing, rehydration, or reconstitution prior to its use, which is a key feature for clinical use. Such a bone paste could replace the bone substitutes that are in powder form, particularly for the irregular or hard-to-reach areas frequently encountered in oral and maxillofacial bone surgery. However, aside from its handling and physical characteristics, proof of concept of its bone regenerative capacity should be established prior to its clinical transfer. In this context, the present work aimed to provide a thorough assessment of the bone healing capacity of this allogeneic bone paste compared with an allogeneic particular bone graft before its partial demineralization.

The purpose of this study was hence to assess the effects of the bone paste in vitro (1) on the attachment and osteoblastic commitment of primary human mesenchymal stromal cells from bone marrow (hBM-MSCs), (2) on the polarization of primary human monocytes isolated from circulating blood, and (3) to test and validate the in vivo capacity of this innovative bone paste to support and promote bone healing in two preclinical models: a guided bone regeneration (GBR) and a critical size defect (CSD) model in rat calvaria to mimic intramembranous bone healing of oral and maxillofacial defects.

## Results

### Attachment and osteoblastic commitment of hBM-MSCs

**Figure 1:**
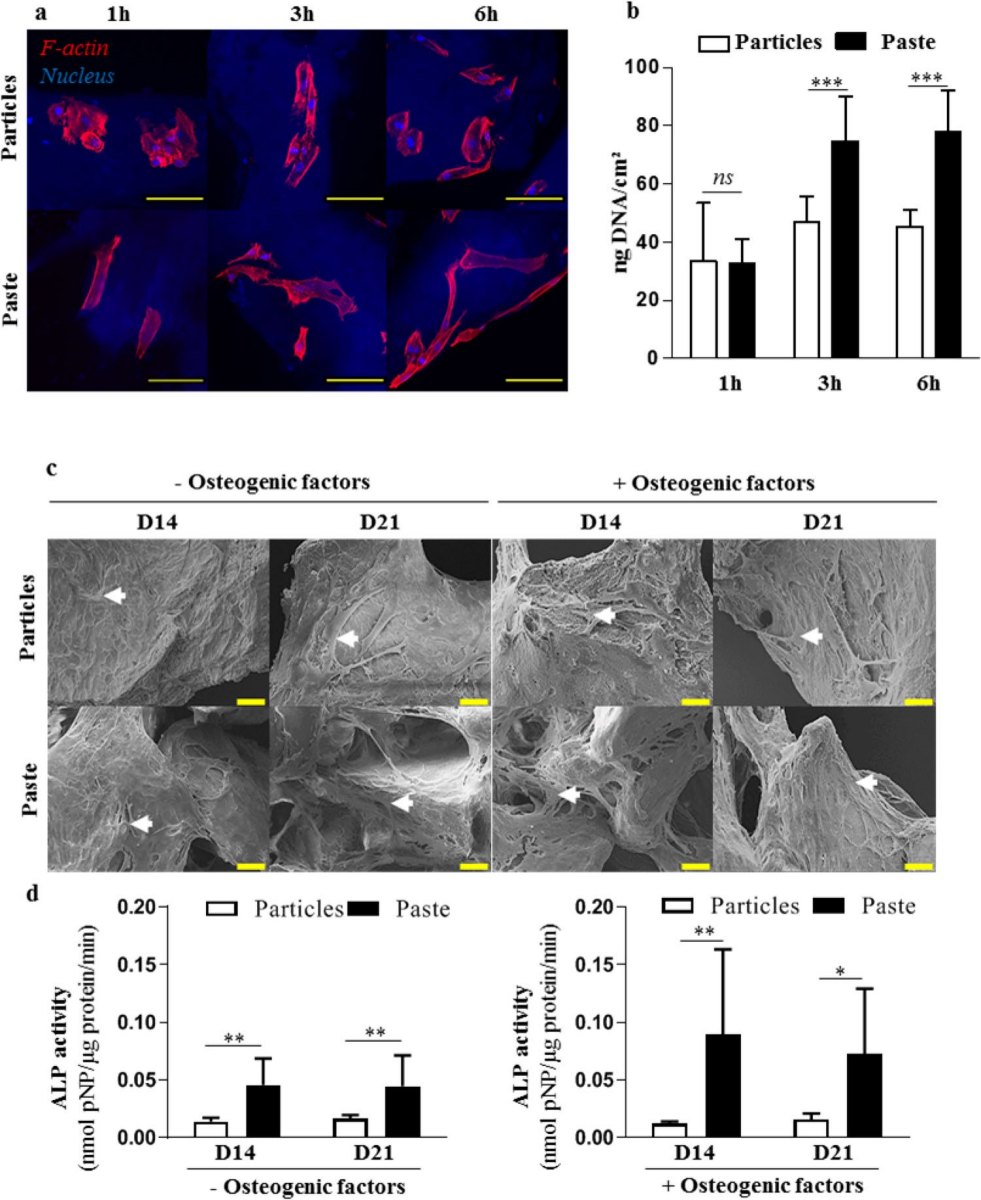
Attachment and osteoblastic commitment of hBM-MSCs cultured in contact with the particular bone graft or the bone paste. (**a**) Representative confocal images of stained hBM-MSCs. Red (phalloidin-AF568): F-actin, blue (Hoechst): nucleus. The particular bone graft and the bone paste were visible as a result of their blue auto fluorescence. Scale bars: 100 µm. (**b**) Quantification of the total DNA in hBM-MSCs in contact with the particular bone graft or the bone paste. (**c**,**d**) SEM images and ALP activity in lysates of hBM-MSCs grown in contact with the particular bone graft or bone paste with or without osteogenic factors for 14 and 21 days. Scale bars: 20 µm. The results are expressed as means ± the SD (N = 3, n = 3; *p < 0.05, **p < 0.01 ***p < 0.001).

The attachment of hBM-MSCs was first investigated after short periods (1, 3, and 6 h) of contact with the particular bone graft or the bone paste. After 1 h of contact, the cells on the surface of the particular bone graft exhibited a round shape, while they had an elongated and spread-out shape on the surface of bone paste. Interestingly, at 3 h and 6 h, all of the cells exhibited an elongated and spread-out shape (Fig. 1a) irrespective of the graft. These data strongly suggest that the bone paste allows faster cell adhesion compared to the particular bone graft. Quantification of the DNA content of the adherent cells revealed no difference after 1 h of contact. However, after 3 and 6 h of contact, the DNA content was significantly higher (60% and 70%, respectively) for the cells cultured in contact with the bone paste compared to the cells in contact with the particular bone graft (Fig. 1b).

To determine whether the particular bone graft or the bone paste may affect the osteoblastic differentiation of hBM-MSCs, the cells were cultured in contact with the bone grafts in culture medium with or without osteogenic factors for 14 and 21 days. As seen by the SEM analyses, the osteogenic factors did not influence the colonization of the particular bone graft or the bone paste by the hBM-MSCs. Under all of the culture conditions, the cells had elongated cellular bodies and cytoplasmic expansions (Fig. 1c, white arrows). Of interest, the cohesion between the bone paste allowed the cells to create a network from particle to particle, with or without osteogenic factors, at all of the time points (Fig. 1c). It is also worth noting that this phenomenon was not observed with the cells cultured in contact with the particular bone graft. Finally, to evaluate the osteogenic commitment of the hBM-MSCs cultured in contact with the particular bone graft or the bone paste, the ALP activity was measured in cell lysates, and it was found to be significantly higher in the cells cultured in contact with the bone paste compared to the cells in contact with the particular bone graft: 3.3 and 2.7 times higher, respectively, in the absence of osteogenic factors and 7.3 and 4.6 times higher, respectively, in the presence of osteogenic factors after 14 and 21 days of culture (Fig. 1d).

### Intramembranous bone healing in a GBR model

**Figure 2:**
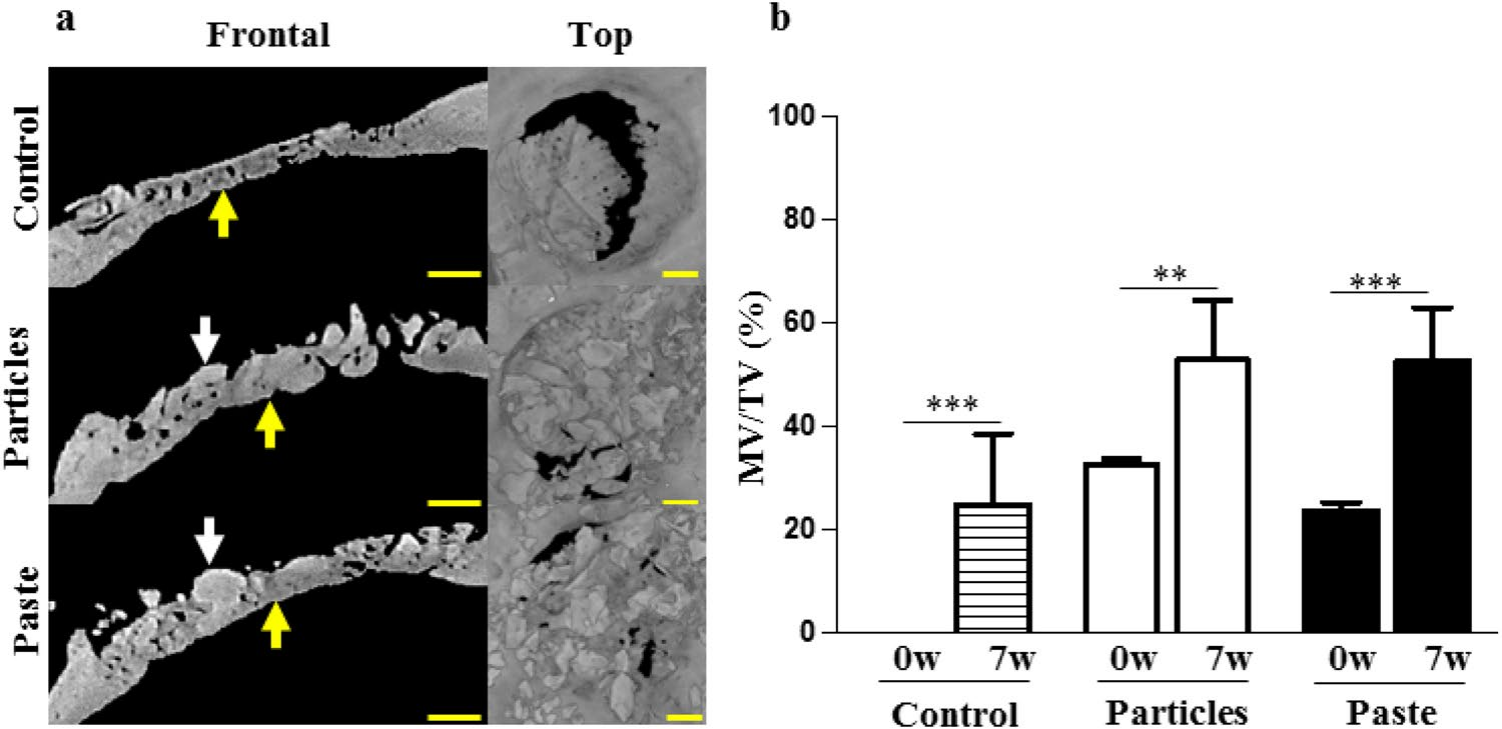
Rat calvaria healing 7 weeks after surgery in a guided bone regeneration model. (**a**) Representative images of the reconstructed µ-CT acquisitions (yellow arrows: newly formed bone, white arrows: grafts). The control represents the defects with the GBR membrane without bone grafts (unfilled defects). Scale bars: 1 mm. (**b**) Three-dimensional µ-CT quantitative analysis of the mineral volume (MV) within the tissue volume (TV) in the defects at the time of surgery (0w, baseline) and 7 weeks (7w) after surgery. The results are expressed as means ± the SD (n = 6) *p < 0.05, **p < 0.01, ***p < 0.001).

To mimic clinical indications of guided bone regeneration (e.g., alveolar socket preservation), the ability of the bone paste to support bone healing was first assessed in a GBR model in rat calvaria. The µ-CT analyses of the control defects at 7 weeks post-surgery revealed a significant degree of centripetal bone ingrowth from the edges of the defects (Fig. 2a), as expected in this GBR model. In the grafted defects, homogenous bone ingrowth (Fig. 2a, yellow arrows) was observed, but the particular bone graft and the bone paste were still visible in the defects (Fig. 2a, white arrows). The three-dimensional quantification of the MV/TV, increasing over time, indicated that new bone formation occurred in the control and in the grafted defects (Fig. 2b). However, the increase in the MV/TV between 0 and 7 weeks was greater in the group grafted with the bone paste than in the group grafted with the particular bone graft (2.2 vs. 1.6-fold, respectively).

**Figure 3:**
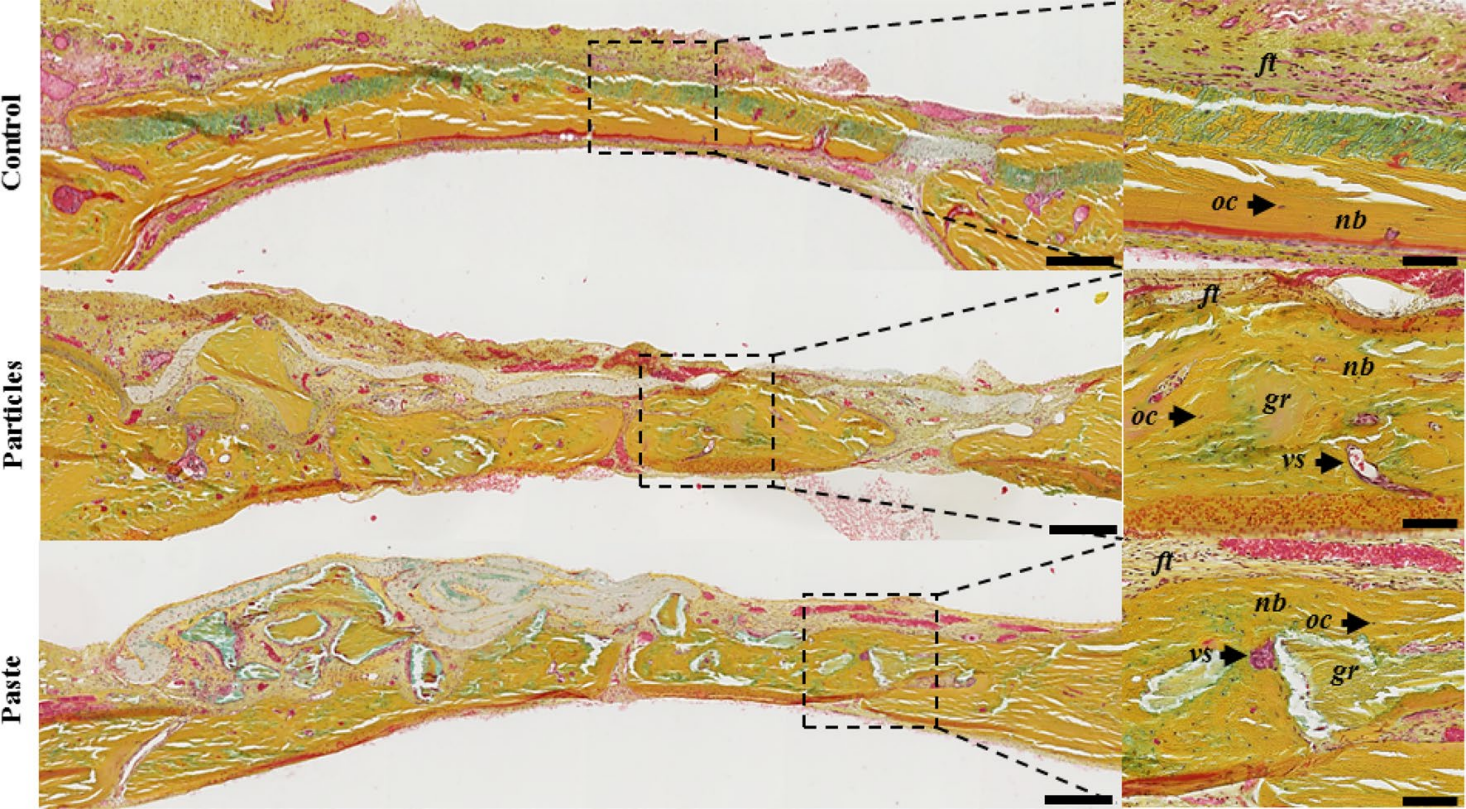
Rat calvaria healing 7 weeks after surgery in a guided bone regeneration model. Representative images of the defects with Movat’s pentachrome staining of undecalcified 7-µm thick frontal sections. The control represents the defects with the GBR membrane without bone grafts (unfilled defects). Scale bars: 250 µm for the histological full defects and 100 µm for the histological magnification (dashed square). Dark yellow: collagen in the bone tissue, light red: cytoplasms, dark red: osteoid borders, brown: nuclei, *ft*: fibrous tissue, *oc*: osteocytes, *nb*: newly formed bone, *vs:* blood vessels, *gr*: grafts.

In all of the defects, the histological observations revealed the collagen-rich and layer-organized nature of the newly formed bone (yellow matrix), with numerous osteocytes, that was identical to the native bone outside the defects (Fig. 3). These observations confirmed the µ-CT analyses, with significant bone formation after 7 weeks in the control defects, yet without continuous newly formed bone tissue (i.e., full closure). In the defects implanted with the particular bone graft and the bone paste, the grafts were perfectly osteointegrated and still visible (Fig. 3, *gr*) and recognizable due to their shape and the absence of osteocytes inside the grafts.

### Intramembranous bone healing in a CSD model

**Figure 4:**
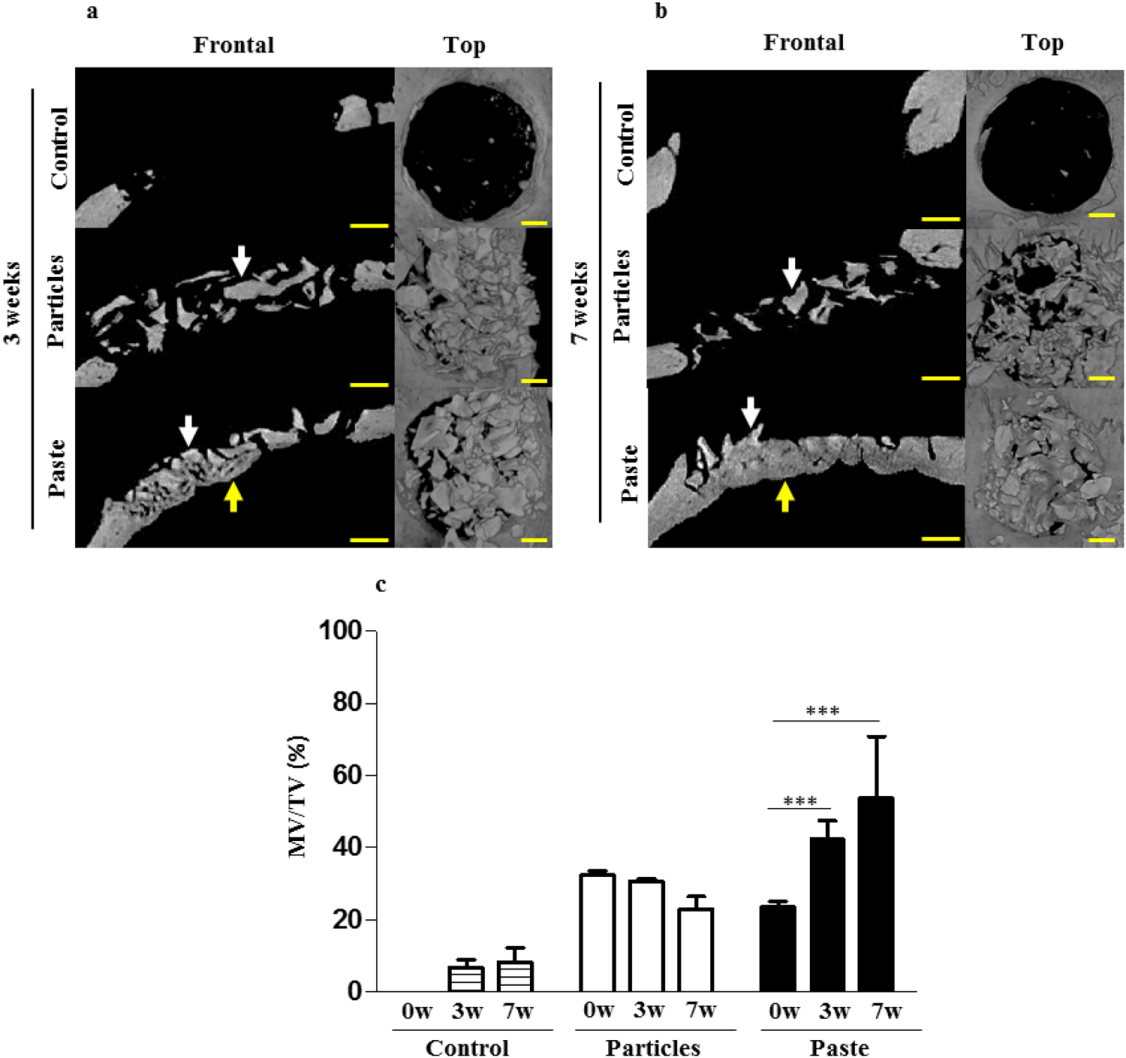
Rat calvaria healing at 3 and 7 weeks after surgery in a critical size defect model. (**a**,**b**) Representative images of the reconstructed µ-CT acquisitions (yellow arrows: newly formed bone, white arrows: grafts). The control represents the unfilled defects. Scale bars: 1 mm. (**c**) Three-dimensional µ-CT quantitative analysis of the mineral volume (MV) within the tissue volume (TV) in the defects after 0 weeks (baseline), 3, and 7 weeks of regeneration. The results are expressed as means ± the SD (n = 6) **p < 0.01, ***p < 0.001).

The intrinsic bone-forming ability of the bone paste was then tested using a challenging CSD model of intramembranous bone healing. Based on the reconstructed µ-CT images, it appeared that no newly formed bone was observable in the control defects or filled with the particular bone graft at 3 and 7 weeks post-surgery (Fig. 4a,b white arrows). This was confirmed by the MV/TV quantifications (Fig. 4c). By contrast, in the defects filled with the bone paste, newly formed bone was clearly visible after 3 and 7 weeks (Fig. 4a,b yellow arrows), thus indicating healing of the defects in the presence of the bone paste.

**Figure 5:**
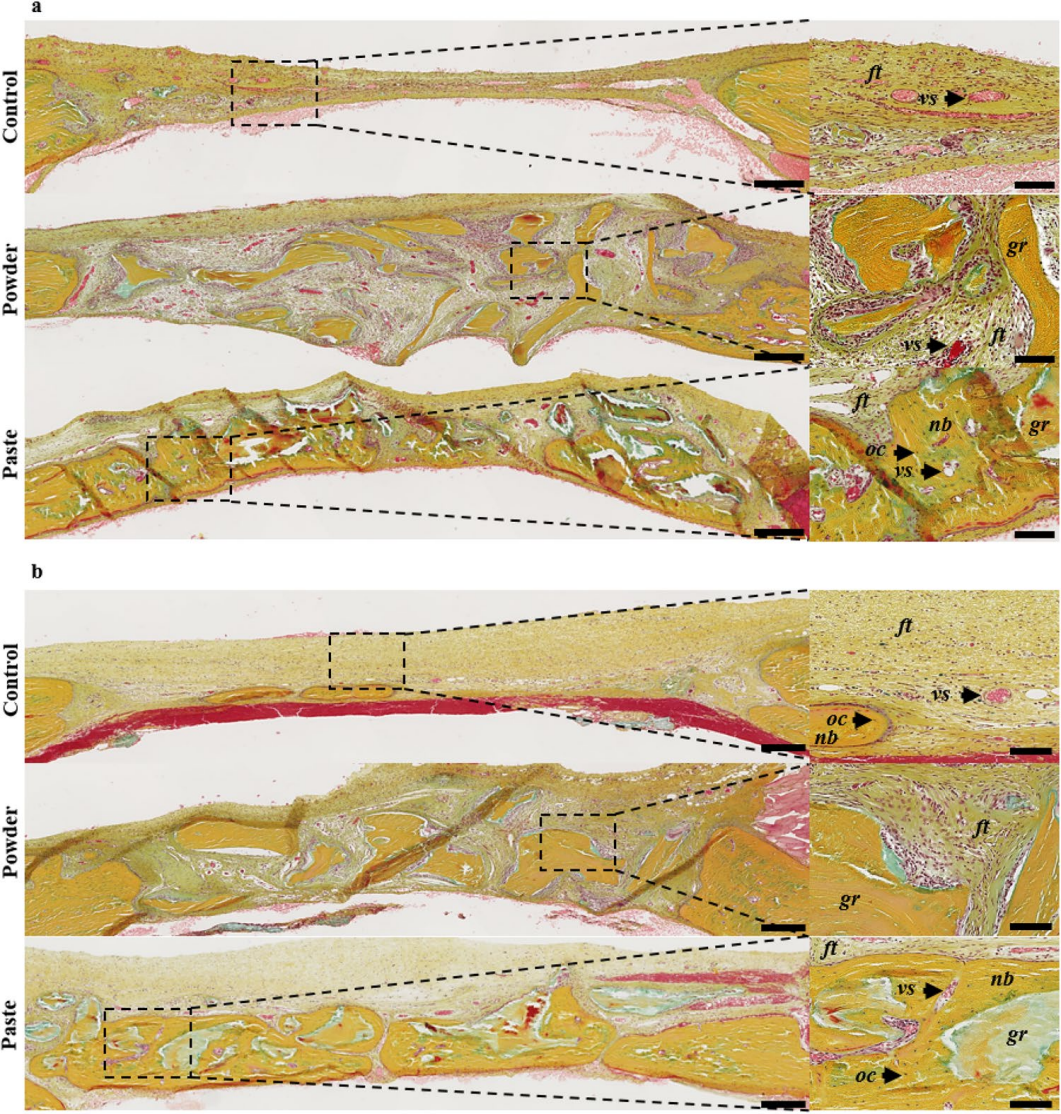
Rat calvaria healing at 3 and 7 weeks after surgery in a critical size defect model. Representative images of the defects with Movat’s pentachrome staining of undecalcified 7-µm thick frontal section, 3 (**a**) or 7 (**b**) weeks after the surgery. The control represents the unfilled defects. Scale bars: 250 µm for the full defects, 100 µm for the high-magnification image (dashed square). Dark yellow: collagen in the mature bone, light red: cytoplasms, dark red: osteoid borders, brown: nuclei, *ft:* fibrous tissue, *oc*: osteocytes, *nb*: newly formed bone, *vs:* blood vessels, *gr*: grafts.

The histological observations confirmed the radiological analyses and showed that a barely detectable amount of new bone had formed at the edges but not at the center of the control defects at 3 weeks (Fig. 5a) and 7 weeks (Fig. 5b) post-surgery. The same observation was made in the defects filled by the particular bone graft at 3 weeks (Fig. 5a) and 7 weeks (Fig. 5b) post-surgery. In this group, three weeks after the implantation, the soft tissue appeared broadly vascularized and highly cellularized in close proximity to the particular bone graft (Fig. 5a). The surface of these particular bone graft was light blue, probably due to the fact that the surfaces had lost their collagen content (which appeared yellow by Movat’s pentachrome staining). Seven weeks after the particular bone graft implantation, the soft tissue appeared to be less vascularized and less cellularized compared to the defects observed 3 weeks after the implantation. On the other hand, the defects filled with the bone paste exhibited a substantial proportion of newly formed bone, which was already visible 3 weeks post-surgery (Fig. 5a, *nb*). Despite the fact that the newly formed bone was distributed heterogeneously in the defects 3 weeks after the surgery, lamellar bone structures with numerous osteocytes and a number of blood vessels could be observed (Fig. 5a, *nb*, *vs*). Of interest, the bone paste had become fully integrated in the regenerated bone 7 weeks after the surgery (Fig. 5b, *gr*), as discernible in the defects with their characteristic acellular mineralized inner core and demineralized outer part.

At this time point, the bone regrowth in the defects filled with the bone paste was much more abundant and homogeneous compared to at 3 weeks (Fig. 5b), with a limited amount of soft tissue. Moreover, the regenerated bone (Fig. 5b, *nb*) exhibited a typical lamellar osseous structure, with numerous osteocytes (Fig. 5b, *oc*) and vasculature reminiscent of that observed in native bone tissue outside the grafted area.

### Monocyte/macrophages phenotypic changes

Having demonstrated that the bone paste had greater regenerative properties than the particular bone graft in two complementary models of intramembranous bone healing, we sought to determine whether monocytes/macrophages may play a pivotal role in the discrepancy found in the regenerative efficiency of the two types of bone grafts.

**Figure 6:**
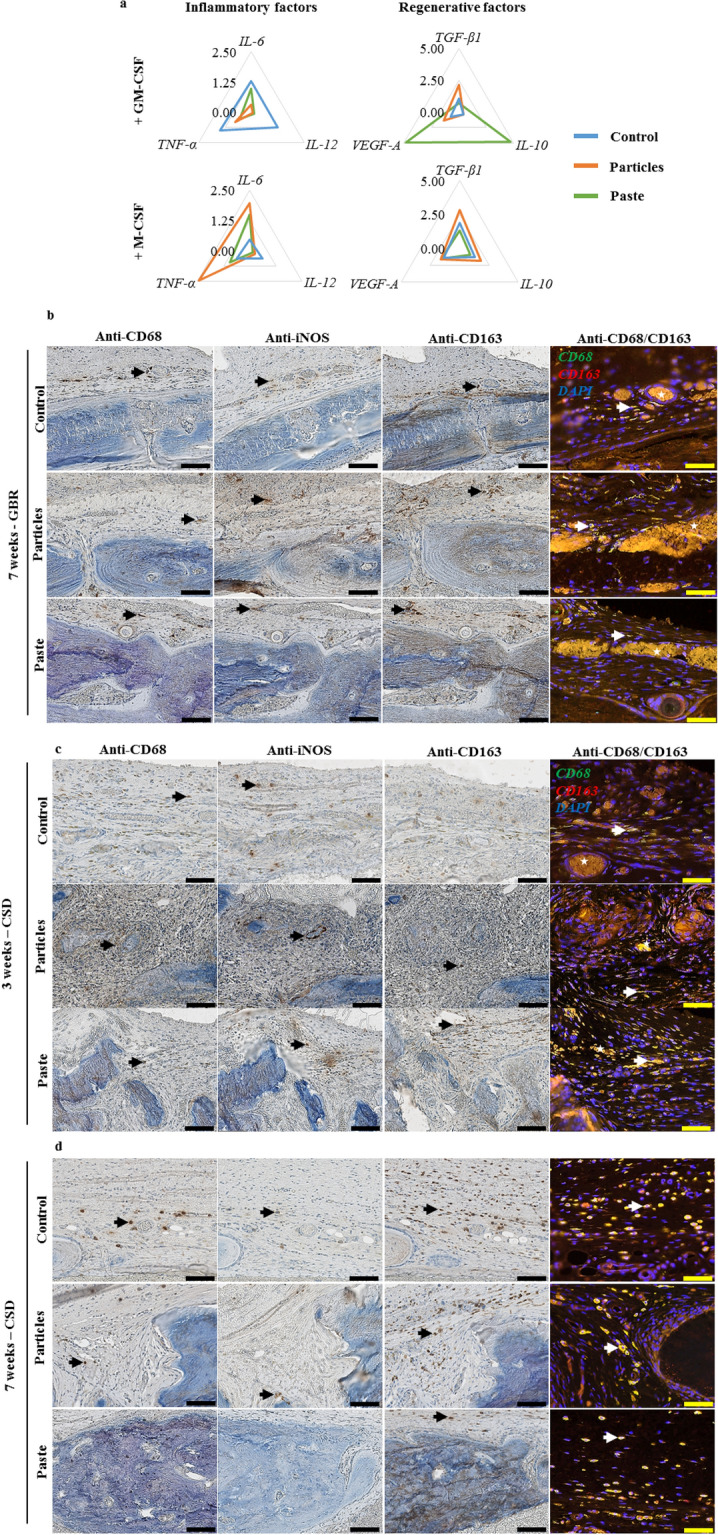
Effect of the particular bone graft and the bone paste on monocyte/macrophage polarization. (**a**) mRNA expression in human monocytes cultured in the presence of GM-CSF or M-CSF for 3 days in contact with the particular bone graft or the bone paste. The Y-axis represents the mean relative mRNA expression level of the specified genes (N = 3). Representative immunohistochemical and immunofluorescent stainings on successive undecalcified 7-µm thick sections, (**b**) in the GBR model after 7 weeks, and (**c**) in the CSD model after 3 and 7 weeks. Immunohistochemical stainings were performed for CD68, iNOS, CD163, and immunofluorescence with double labeling of CD68 (green)/CD163 (red) and DAPI (blue) for the nuclei. For single labeling, positive cells were stained with a brown precipitate (black arrow), for double labeling, double-positive cells appeared as light yellow (white arrow). Typical auto fluorescent red blood cells were visible (white stars). Scale bars: 100 µm for the immunohistochemistry, 50 µm for the immunofluorescence images.

When cultured under pro-inflammatory conditions, the macrophages in contact with the particular bone graft expressed lower levels of *TNF-α, IL-6,* and *IL-12* mRNA level (classical markers of inflammation) compared to the control without grafts (Fig. 6a). The macrophages cultured in contact with the bone paste also expressed lower levels of *TNF-α, IL-6,* and *IL-12* compared to the control. However, they exhibited higher *VEGF-A* and *IL-10* mRNA expression (classical pro-regenerative markers), thereby suggesting a substantial pro-regenerative phenotype (Fig. 6a). On the other hand, when cultured under pro-regenerative conditions, the macrophages exhibited no change in *VEGF-A*, *TGF-β,* or *IL-10* mRNA expression when in contact with the particular bone graft or the bone paste compared to the control, thus indicating that the pro-regenerative phenotype of the macrophages was maintained in these conditions (Fig. 6a). However, the macrophages in contact with the bone paste also expressed lower *IL-6* and *TNF-α* mRNA levels than those in contact with the particular bone graft, supporting the role of the bone paste to enhance the regenerative phenotype of the macrophages (Fig. 6a).

To further determine whether this in vitro observation may have a degree of in vivo relevance, the phenotype of the macrophages was analyzed in the calvarial defects by immunohistochemical labeling [CD68 (total macrophages), iNOS (pro-inflammatory macrophages), and CD163 (pro-regenerative macrophages)].

In the GBR model, CD68^+^, iNOS^+^, or CD163^+^ cells (black arrows), as well as CD68^+^/CD163^+^ cells (white arrows) were found in the control group as well as in the vicinity of the particular bone graft (Fig. 6b). However, in the group grafted with the bone paste, while CD68^+^, CD163^+^, and CD68^+^/CD163^+^ cells were found in the regenerated tissue, few iNOS^+^ cells were observed. This strongly suggests that the macrophages had largely assumed a pro-regenerative phenotype 7 weeks after the implantation.

In the CSD model, after 3 and 7 weeks, CD68^+^, iNOS^+^, CD163^+^, or CD68^+^/CD163^+^ cells were present in the control group (Fig. 6c). In the grafted groups, our data suggest that the macrophages had a more pronounced pro-regenerative engagement in the defects filled with the bone paste than with the particular bone graft. This difference was clearly observable after 3 weeks of regeneration. In the defects filled with the particular bone graft, CD68^+^ and iNOS^+^ cells could be seen, while CD163^+^ and CD68^+^/CD163^+^ cells were hard to discern. Conversely, in the defects filled with the bone paste, CD68^+^, CD163^+^, CD68^+^/CD163^+^ cells were present, and few iNOS^+^ cells could be seen. After 7 weeks of regeneration, the unfilled defects and the defects filled with the particular bone graft contained CD68^+^, iNOS^+^, CD163^+^, and CD68^+^/CD163^+^ cells. At this time point, the defects grafted with bone paste were filled with newly formed bone, and hence few monocytes/macrophages could be seen in the defects. Indeed, only CD163^+^ and CD68^+^/CD163^+^ cells were present in the soft tissue in the regenerated defects (Fig. 6d).

## Discussion

Allogeneic bone grafts are a suitable and widely used alternative to ABGs in oral and maxillofacial bone graft procedures. However, allogeneic bone is mainly used in powder or block forms, and its lack of versatility and adaptability limits its use in irregular or hard-to-reach areas. In this work, the regenerative potential of an easy-to-handle and ready-to-use moldable allogeneic bone graft in a paste form made of partially demineralized bone allograft was assessed, with the aim of supplanting the use of allogeneic particular bone grafts in oral and maxillofacial bone grafting procedures.

Our data first of all demonstrated better attachment of hBM-MSCs to the surface of the bone paste compared to the particular bone graft. The greater attachment could be due to the partial demineralization and heating of the bone paste, which modifies the surface of the graft and hence also the cell-material interaction. Although further analyses (e.g., particle topography, kinetics of focal adhesion formation) are required to properly characterize the nature of the interactional changes, it has been reported that the denaturation of collagen can expose cryptic adhesion sites and RGD (Arg-Gly-Asp) integrin-binding sites that promote the adhesion of hBM-MSCs^13–18^. Additionally, the bone paste was found to remain cohesive for up to 21 days in an aqueous medium, and it promoted the osteogenic commitment of hBM-MSCs. This latter property is of great relevance for bone regeneration since the bone paste could act as a cohesive scaffold in the bone defects. This may lead to better osteointegration and osteoconduction than with independent and non-demineralized bone grafts. The higher ALP activity observed in hBM-MSCs cultured in contact with the bone paste compared to the particular bone graft may be due to the different interaction between the cells and the bone grafts, and/or to the presence of a cellular network at the surface of the bone paste, thereby enhancing the osteogenic commitment of hBM-MSCs. Moreover, type I collagen is thought to drive osteogenic fate, and the exposure of RGD binding sites is known to enhance the osteogenic phenotype of mesenchymal stromal cells^18–21^.

These positive in vitro effects of the bone paste on the mesenchymal progenitors were also confirmed through the regenerative potential of the bone paste investigated in a GBR and a CSD model in rat calvaria. In light of the similar organization and ossification between the cranial and maxillary bones, these calvarial models are widely used for preclinical studies focused on the development of bone substitutes for oral and maxillofacial surgeries^22^. Additionally, this operating site allows easy and rapid examination of the bone healing properties of the grafted materials^23–25^. Moreover, such regenerative properties of bone graft have to be investigated in GBR and CSD models, as they are representative of bone healing in techniques that are clinically used in several oral and jaw surgery indications (e.g., sinus lift, socket preservation). However, the atrophic environment of the rat calvaria (poor vasculature and bone marrow) is a challenge when it comes to achieving bone regeneration.

The covering membrane in the GBR model prevents non-osteoblastic cells from migrating from the surrounding soft tissues, thereby indirectly favoring invasion of the bone defects by osteoprogenitors, which improves the bone regeneration^26–28^. In the CSD model, as the bone regeneration is slower compared to the GBR model, an intermediate time point, 3 weeks in addition to 7 weeks of regeneration, was chosen to investigate the early events that are crucial for bone regeneration in these challenging conditions.

The bone regeneration observed in the defects filled with the bone paste in the GBR model validated the ability of this graft to support bone healing. Moreover, the more pronounced increase in the MV/TV between 0 and 7 weeks in the group grafted with the bone paste compared to the group grafted with the particular bone graft can be explained either by the lower MV/TV of the bone paste at the implantation, due to the demineralized part of the bone paste, or by a higher amount of newly formed bone**.** In the CSD model, the radiological and histological observations showed that the particular bone graft failed to support bone repair. The fibrous tissue observed 3 weeks post-implantation suggests that a fibrous encapsulation of the graft occurred, thereby preventing the bone formation. Longer time points would have been necessary to evaluate the healing properties of the particular bone graft in this CSD model.

Nonetheless, the absence of calvarial bone healing has been observed for similar time points, 7 weeks post implantation, with alternative bone filling materials such as synthetic biphasic calcium phosphate^29,30^ or xenogeneic bone^31^ which are widely clinically used. Those particular bone grafts substitutes are known to be mainly osteoconductives, which is not sufficient to allow a bone regrowth in an atrophic site (i.e., low vasculature and the absence of bone marrow) such as calvaria. The particular bone graft used in this study (i.e., cleaned, grinded and sterilized bone tissue) displayed insufficient bone healing properties to promote bone regrowth in the CSD model. Since the particular bone graft had no effect on cell viability (Sup. Fig. 2), the lack of new bone formation in the particular bone graft-filled defects is likely to be related to the low self-healing capacity of the calvarial bone.

In contrast with the particular bone graft, the bone paste induced bone regeneration. This effect is probably not due to the release of growth factors^32,33^ from the demineralized bone matrix, since the heat treatment that the bone graft was subjected to has been reported to denature growth factors^34^. Therefore, we hypothesize that bone regeneration could be induced by the cell-particle interaction and cellular network that is known to promote osteoblastic differentiation and to foster bone regeneration^35,36^. Additionally, the cohesive nature of the bone paste may prevent it from leaking out of the defect, which is notably advantageous in irregular and hard-to-reach areas, which are common in oral and maxillofacial surgery.

Lastly, we investigated the phenotype of the macrophages in contact with the particular bone graft and the bone paste, as these cells are among the first to invade bone defects and interact with grafted materials. Indeed, pro-inflammatory “M1-like” macrophages in early immune responses^37–39^ and pro-regenerative “M2-like” macrophages in the later stages of bone healing^40–42^ are both critically required for proper bone regeneration. The macrophages in contact with the bone paste expressed lower *IL-12* and higher *IL-10* mRNA levels than those in contact with the particular bone graft. This result supports the hypothesis that an “M1-like” to “M2-like” phenotypic switch known to promote inflammation resolution, wound repair, and bone healing^43–46^ was induced by the bone paste. In addition, the increase in *VEGF-A* mRNA expression in the macrophages in contact with the bone paste may also contribute to acceleration of the bone repair process, since angiogenesis plays a pivotal role in bone growth and healing^47–51^.

Our overall immunohistochemical observations also strongly suggest that the bone paste either accelerated the on-site monocyte/macrophage recruitment and/or their “M1-like” to “M2-like” phenotypic switch compared to the particular bone graft. Indeed, the small number of iNOS^+^ cells and the abundance of CD163^+^ and CD68^+^/CD163^+^ cells in the defects filled with the bone paste suggest a suitable inflammatory environment for bone regeneration, with the polarization of the macrophages towards a pro-regenerative phenotype. As the initial inflammation phase may be shortened in these defects, the mineralization phase of the bone tissue could start earlier as well and thus explain the higher MV/TV after 7 weeks in the defects filled with the bone paste compared to the particular bone graft in both the GBR and the CSD model. This is of notably clinical relevance in oral and jaw surgeries, since shortening of the bone healing time may enhance the speed and stability of osteointegration of dental implants, thereby leading to a reduction of the associated treatments or temporary prostheses that often cause significant discomfort for patients.

## Conclusions

To the best of our knowledge, this study is the first to demonstrate the bone regenerative capacity of a partially demineralized bone graft in a paste form in vitro and in vivo. In this study, we successfully demonstrated that a ready-to-use and easy-to-handle partially demineralized allogeneic bone paste has substantial bone regenerative properties. The ability of the allogeneic bone paste to induce bone formation by mesenchymal progenitor cells and macrophages, as well as its radio-opacity, are key features for clinicians. Moreover, as a ready-to-use bone substitute, this paste does not require any additional compounds prior to its use, which is an advantage when considering both regulatory approval and potential clinical indications. Our promising preclinical data have recently led us to initiate an interventional randomized clinical trial for pre-implant guided bone regeneration in oral surgery (NCT04141215).

## Materials and methods

### Process to obtain the bone paste

The allogeneic particular bone graft and bone paste were obtained from human donors who had provided their informed consent, and all of the protocols were approved by the French ethical committee “Comité de Protection des Personnes” (CPP), in accordance with the 1975 Helsinki declaration and its subsequent amendments. The bone allografts were harvested during total hip replacement surgeries and donated to the BIOBank bone bank (Presles-en-Brie, France), under the authorization of the “Agence Nationale de Sécurité du Médicament et des produits de santé” (ANSM, n°FR07703T-19-01) according to the relevant French regulations and ethical standards. Cleaning of the femoral heads was ensured by supercritical CO_2_ lipid extraction followed by successive immersions in H_2_O_2_, NaOH, and EtOH, leaving the bone matrix defatted^52^, virally inactivated^53,54^, and unaltered^55–57^. The cleaned femoral heads were crushed to generate the particular bone graft (0.3 to 1 mm in diameter, Sup. Fig. 1, Sup Vid. 1). The particular bone graft was either sterilized by 25 kGy gamma irradiation and used as an internal comparator, or it was processed into a paste form. For this purpose, the particular bone graft was partially demineralized with HCl, then hydrated with water to make it an extrudable and moldable preparation (Sup. Fig. 1, Sup Vids. 2 and 3), and heated (121 °C) to ensure partial transformation of the outer demineralized collagen of the particles into gelatin and simultaneous sterilization of the entire bone graft^18^. All of the particular bone graft and the bone paste samples were made from pooled donor material in order to limit the intra-donor variability.

The bone and paste were imaged with transmitted light (Leica DM12 LED, Wetzlar, Germany) or reflected light (Leica M125). For all of the X-ray micro-computed tomography (µ-CT) acquisitions, the samples were scanned under the following conditions: resolution: 8 µm/px, single 360° scan, rotation: 0.710°, averaging frames: 4 (SkyScan-1072, Bruker, Billerica, USA). The three-dimensional datasets were reconstructed with the NRecon software (Micro Photonics Inc, Allentown, USA). For the scanning electron microscopy (SEM) imaging, the samples were sputtered with a gold plasma (Sputter Coater Desk Denton Vacuum, Moorestown, USA), and the images were taken using an electron microscope (Zeiss, Oberkochen, Germany) with a secondary electron detector set at 10 kV.

### Analysis of hBM-MSCs attachment and osteoblastic commitment

Human BM-MSCs from two independent donors (one male and one female, PromoCell, Heidelberg, Germany) were amplified in PromoCell Growth Medium supplemented with 1% penicillin–streptomycin (P/S) (Invitrogen, Paisley, UK) in a humidified atmosphere at 37 °C, 5% CO_2_ (Air Liquide, Paris, France). The medium was changed every other day. The cells were used between passage 2 and 5.

To measure the attachment of hBM-MSCs to the particular bone graft and the bone paste, the grafts (0.125 cc/well, 66 and 93 mg, respectively) were spread on the bottom of 24-well plates (Ultra-Low Attachment Surface, Corning). One milliliter of culture medium (DMEM/Ham’s F12 (1:1), high glucose, GlutaMAX (Thermo Fisher Scientific) with 15% fetal calf serum (FCS, Dominique Dutscher, Brumath, France) and 1% P/S) was added to each well and the plates were incubated in a humid atmosphere at 37 °C, 5% CO_2_ for 24 h. The medium was then removed and 1 × 10^5^ hBM-MSCs were seeded in each well. After 1 h, 3 h, and 6 h of contact between the hBM-MSCs and the particular bone graft or the bone paste, the cells were extensively rinsed with 1X phosphate buffered saline (PBS) at 37 °C to remove the non-adherent cells. The DNA content of remaining adherent cells in contact with the particular bone graft or the bone paste was measured with Quant-iT™ PicoGreen dsDNA Reagent (Thermo Fisher Scientific) according to the manufacturer’s instructions and then normalized to the surface of the bone grafts measured by µ-CT as mentioned in subsection 2.1.

For the confocal imaging, the cells were fixed in 4% paraformaldehyde (PFA) for 20 min and then permeabilized with Triton X-100 (1% in 1X PBS) for 10 min at room temperature. The cells were then labeled with 1:200 Alexa Fluor 568 phalloidin (Thermo Fisher Scientific) in 1X PBS at room temperature for 1 h, and the nuclei were stained with 1:50,000 Hoechst 33258 (Thermo Fisher Scientific) in 1X PBS for 15 min at room temperature. The cell samples were imaged using a confocal microscope (A1RS, Nikon, Champigny sur Marne, France).

To assess the ability of the particular bone graft and the bone paste to support osteogenic commitment, the level of alkaline phosphatase (ALP) activity was measured in cell lysates. The particular bone graft and the bone paste were placed in low-binding 24-well plates as described above, and 1 × 10^5^ hBM-MSCs were seeded in each well. After 48 h, the medium was replaced with culture medium with or without osteogenic factors (50 µM ascorbic acid, 10 mM β-glycerophosphate, and 100 nM dexamethasone (Thermo Fisher Scientific)). The medium was changed every other day. The ALP activity was measured after 14 or 21 days with an Alkaline Phosphatase Substrate kit (Bio-Rad, Hercules, USA) and normalized to the total protein content, measured using a BCA protein assay kit (Thermo Fisher Scientific) according to the manufacturer’s instructions.

### Monocyte/macrophage polarization

Monocytes and human serum (HS) were obtained from the clinical transfer platform of Nantes Hospital (PF DTC, CIC 0503). Monocytes were seeded in 12-well plates at a density of 1.25 × 10^6^ cells in 2.5 mL of culture medium (RPMI 1640, Thermo Fisher Scientific) with 8% HS, 2 mM l-glutamine, 100 IU/mL penicillin, and 0.1 mg/mL streptomycin. To induce pro-inflammatory “M1-like” macrophages, the medium was supplemented with GM-CSF (granulocyte–macrophage colony-stimulating factor) at 20 ng/mL (CellGenix GmbH, Freiburg im Breisgau, Germany) and to induce pro-regenerative “M2-like” macrophages, the medium was supplemented with M-CSF (macrophage colony-stimulating factor) at 50 ng/mL (Isokine, Kopavogur, Iceland), for 3 days, as previously described^58,59^. The cells were cultured in contact with the particular bone graft, the bone paste (0.125 cc/well, 66 and 93 mg, respectively), or in the absence of the graft as a control. After 3 days, lipopolysaccharide (LPS) (Sigma-Aldrich, St. Louis, USA) at 200 ng/mL was added to each well and the total RNA was extracted after 6 h.

To investigate the monocyte/macrophage polarization in contact with the particular bone graft and the bone paste, real-time PCR was used to measure the expression levels of genes encoding specific markers of the monocyte/macrophage phenotype. Total RNA was prepared with TRIzol Reagent (Thermo Fisher Scientific), followed by Nucleospin XS RNA columns (Macherey–Nagel, Hoerdt, France) according to the manufacturer’s instructions. A 0.5 μg quantity of the total RNA was reverse transcribed and analyzed using a Bio-Rad CFX96 detection system using SYBR Select Master Mix (Thermo Fischer Scientific). The relative mRNA expression was normalized to the expression of the *B2M* and *PPIA* housekeeping genes and calculated using the 2^−∆Ct^ method with Bio-Rad CFX Manager software. The primer sequences and the names of the target and the housekeeping genes are indicated in Table 1.

**Table 1:** Targets, names, and sequences of the primers used for the real-time PCR analysis of mRNA expression in human monocytes/macrophages.

Gene	Common name	Primer names/sequence	Supplier
*IL-12*	Interleukin-12	QT00000364	Qiagen
*IL-6*	Interleukin-6	QT00083720	Qiagen
*TNF-α*	Tumor necrosis factor-alpha	QT01079561	Qiagen
*TGF-β*	Transforming growth factor-beta	QT00000728	Qiagen
*IL-10*	Interleukin-10	QT00041685	Qiagen
*VEGF-A*	Vascular endothelial growth factor-A	F: 5′ GCTGTCTTGGGTGCATTGGA	Eurofins
		R: 5′ ATGATTCTGCCCTCCTCCTTC	
*B2M*	Beta2-microglobulin	F: 5′ CCTGGAGGCTATCCAGCGTA	Eurofins
		R: 5′ GGATGACGTGAGTAAACCTGAATCT	
*PPIA*	Peptidyl prolyl isomerase A	F: 5′ GTCAACCCCACCGTGTTCTT	Eurofins
		R: 5′ CTGCTGTCTTTGGGACCTTGT	

F: forward, R: reverse.

### In vivo analysis of the bone regeneration in a guided bone regeneration (GBR) and a critical size defect model (CSD)

#### Animals and ethical aspects

The study was carried out in compliance with the ARRIVE guidelines. All methods were carried out in accordance with relevant guidelines and regulations, the institutional guidelines of the European and French Ethics Committee and approved by the local ethics committee (Comité d’Ethique en Experimentation Animale, Pays de la Loire (CEEA.PdL) n°06, agreements n° 2016111513349765/8560 and n°201802021521/15410). Seven-week-old syngeneic male Lewis rats were purchased from an approved breeder (Charles River, Écully, France). A concerted effort was made to minimize physical and psychological suffering and to reduce the number of animals used.

#### Surgical procedures for the GBR and CSD models

All of the veterinary medicines were purchased from Centravet (Dinan, France). The animals were allowed to acclimate for a week at the animal facility before the surgery. The acclimated animals were anesthetized by inhalation of an air/isoflurane mixture (4% isoflurane) in a closed induction chamber. Throughout the surgical procedures, the animals were placed on a heating pad, and the anesthesia was maintained with an air/isoflurane mixture (2% isoflurane) through an inhalation mask. The operating site (the top of the skull) was shaved and the skin was cleaned with sterile water and iodized polyvidone (Betadine). The preoperative analgesia was performed by subcutaneous injection of lidocaine (Xylocaine) at 5 mg/kg on the operative area, and buprenorphine (Buprecare) at 0.02 mg/kg and meloxicam (Metacam) at 1 mg/kg were injected subcutaneously in the back of the rats. The skin and the periosteum were incised and pushed back on the sides, after which full-thickness bilateral paramedian parietal craniotomies were performed using a 5-mm outer diameter trephine (two defects per rat). During the surgery, the defects were abundantly irrigated with saline solution to avoid cauterization of the defect edges by the trephine. The defects were then either left unfilled as a negative control or filled with the particular bone graft (rehydrated with 0.9% NaCl, 0.6 mL/cc for 10 min) or the bone paste.

Two experimental procedures were independently carried out: a GBR and a CSD model. For the GBR model, the defects were covered with a polytetrafluoroethylene membrane. For the CSD model, the defects were left uncovered. For both models, the skin was sutured (5/0, non-absorbable suture (Ethicon, Bridgewater, USA)) and the animals were returned to their cages with water and food ad libitum. The postoperative analgesia was provided by subcutaneous injection of buprenorphine at 0.02 mg/kg twice daily for 3 days and meloxicam at 1 mg/kg mixed in their water supply for 5 days. After 7 weeks for the GBR model, and after 3 or 7 weeks for the CSD model, the animals were euthanized in a CO_2_ chamber. The calvaria were collected using sharp scissors inserted through the *foramen magnum*, and the skulls were cut following the temporal crest to the frontal bone, above the coronal suture. The calvaria were fixed in 4% PFA for 72 h and then stored in 70% ethanol.

### In vivo micro-computed tomography analyses

To quantify the mineral volume (MV) within the tissue volume (TV) (i.e., the bone in the defects), the calvaria were scanned using µ-CT, as mentioned in subsection 2.1. To calculate the MV/TV among the different groups, a hollow cylindrical volume of interest (VOI) of 4.5 mm in diameter and 1 mm in height were selected in the defects. The three-dimensional quantitative results were expressed as MV/TV (%) after 3 or 7 weeks (CTAn software, Bruker, Madison, WI).

### Histological and immunological analyses

Fixed samples were dehydrated by means of a graded series of ethanol baths. Non-decalcified bone specimens were infiltrated and embedded in Technovit 9100 New (Heraeus Kulzer, Les Ulis, France). For each sample, a frontal section was performed through the defects of each explant using a circular diamond saw (Leica, Wetzlar, Germany) and serial 7-μm thick sections were cut using a hard tissue microtome (Polycut, Leica). The sections were stained with Movat’s pentachrome (Thermo Fisher Scientific).

Single immunostainings were performed with three different antibodies (anti-CD68: 1:500 ab125212, anti-CD163: 1:2,000 ab182422, or anti-iNOS (inducible nitric oxide synthase): 1:250 ab15323 (Abcam, Cambridge, UK)). The labeling was visualized with a horse anti-rabbit peroxidase-conjugated antibody (Vector, Burlingame, USA) and 3,3-iaminobenzidine (DAB, Vector). The slides were counterstained with Mayer’s hematoxylin (Microm Microtech, Brignais, France).

For double CD68/CD163 fluorescent labeling, the samples were processed as described above. The incubation with CD163 was followed by incubation with a secondary antibody (Alexa Fluor 568-conjugated goat anti-rabbit 1:200, ab17471). The CD68 was then added, followed by Alexa Fluor 488-conjugated goat anti-rabbit (1:200, ab150081). All of the slides were scanned with a slide reader (NanoZoomer, Hamamatsu).

### Statistical analyses

The statistical analyses were performed using GraphPad Prism 5.0 software (GraphPad, San Diego, USA). Statistical significance was determined using two-way ANOVA followed by Bonferroni's post-test for multiple group comparisons with different variables, or a *t*-test for two-group comparisons. Statistical significance was set at *p* < 0.05. Unless stated otherwise, the experiments were repeated at least three times. The results are presented as means ± the SD.

## Supplementary Information

Supplementary Information 1.

Supplementary Video S1.

Supplementary Video S2.

Supplementary Video S3.
