# Supplementary material for: A partially demineralized allogeneic bone graft: in vitro osteogenic potential and preclinical evaluation in two different intramembranous bone healing models
Source: Sci Rep. 2021 Mar 1;11:4907. doi: 10.1038/s41598-021-84039-6 (PMC7921404; doi:10.1038/s41598-021-84039-6)
Supplement: Supplementary file 1 — Supplementary Information 1. [file 41598_2021_84039_MOESM1_ESM.docx]

A partially demineralized allogeneic bone graft: *in vitro* osteogenic potential and preclinical evaluation in two different intramembranous bone healing models

Pierre Tournier^a,b^, Jérôme Guicheux^c,d^, Arnaud Paré^a,e^, Aymeric Maltezeanu^a^, Thibaut Blondy^f^, Joëlle Veziers^c,d^, Caroline Vignes^a,d^, Manon André^a^, Julie Lesoeur^a,d^, Ana Barbeito^b^, Raphaël Bardonnet^b^, Christophe Blanquart^f^, Pierre Corre^c^, Valérie Geoffroy^a+^, Pierre Weiss^c^*^+^, Alexis Gaudin^c+^

^a^Université de Nantes, Oniris, INSERM, Regenerative Medicine and Skeleton, RMeS, UMR 1229, F-44000, France

^b^BIOBank SAS, Lieusaint, France

^c^Université de Nantes, Oniris, CHU Nantes, INSERM, Regenerative Medicine and Skeleton, RMeS, UMR 1229, F-44000, France

^d^INSERM, UMS 016, CNRS 3556, Structure Fédérative de Recherche François Bonamy, SC3M Facility, CHU Nantes, Université de Nantes, Nantes, F-44042 France

^e^Service de Chirurgie Maxillo-faciale, Plastique et Brulés, Hôpital Trousseau, CHU de Tours, F-37170, France

^f^Université de Nantes, Univ Angers, INSERM, CNRS, CRCINA, F-44000 Nantes, France

+ Authors who contributed equally

* Corresponding author: [pierre.weiss@univ-nantes.fr](mailto:pierre.weiss@univ-nantes.fr); 1 place Alexis Ricordeau 44000 NANTES

[pierre.tournier@univ-nantes.fr](mailto:pierre.tournier@univ-nantes.fr), [jerome.guicheux@univ-nantes.fr](mailto:jerome.guicheux@univ-nantes.fr), [arnaud.pare@univ-tours.fr](mailto:arnaud.pare@univ-tours.fr), [aymeric.maltezeanu@gmail.com](mailto:aymeric.maltezeanu@gmail.com), [thibaut.blondy@gmail.com](mailto:thibaut.blondy@gmail.com), [joelle.veziers@univ-nantes.fr](mailto:joelle.veziers@univ-nantes.fr), [caroline.vignes@univ-nantes.fr](mailto:caroline.vignes@univ-nantes.fr), [manon.andre@univ-nantes.fr](mailto:manon.andre@univ-nantes.fr), [julie.lesoeur@univ-nantes.fr](mailto:julie.lesoeur@univ-nantes.fr), [abarbeito@biobank.fr](mailto:abarbeito@biobank.fr), [rbardonnet@biobank.fr](mailto:rbardonnet@biobank.fr), [christophe.blanquart@univ-nantes.fr](mailto:christophe.blanquart@univ-nantes.fr), [pierre.corre@chu-nantes.fr](mailto:pierre.corre@chu-nantes.fr), [valerie.geoffroy@univ-nantes.fr](mailto:valerie.geoffroy@univ-nantes.fr), [pierre.weiss@univ-nantes.fr](mailto:pierre.weiss@univ-nantes.fr), [alexis.gaudin@univ-nantes.fr](mailto:alexis.gaudin@univ-nantes.fr)

**Supplementary informations**


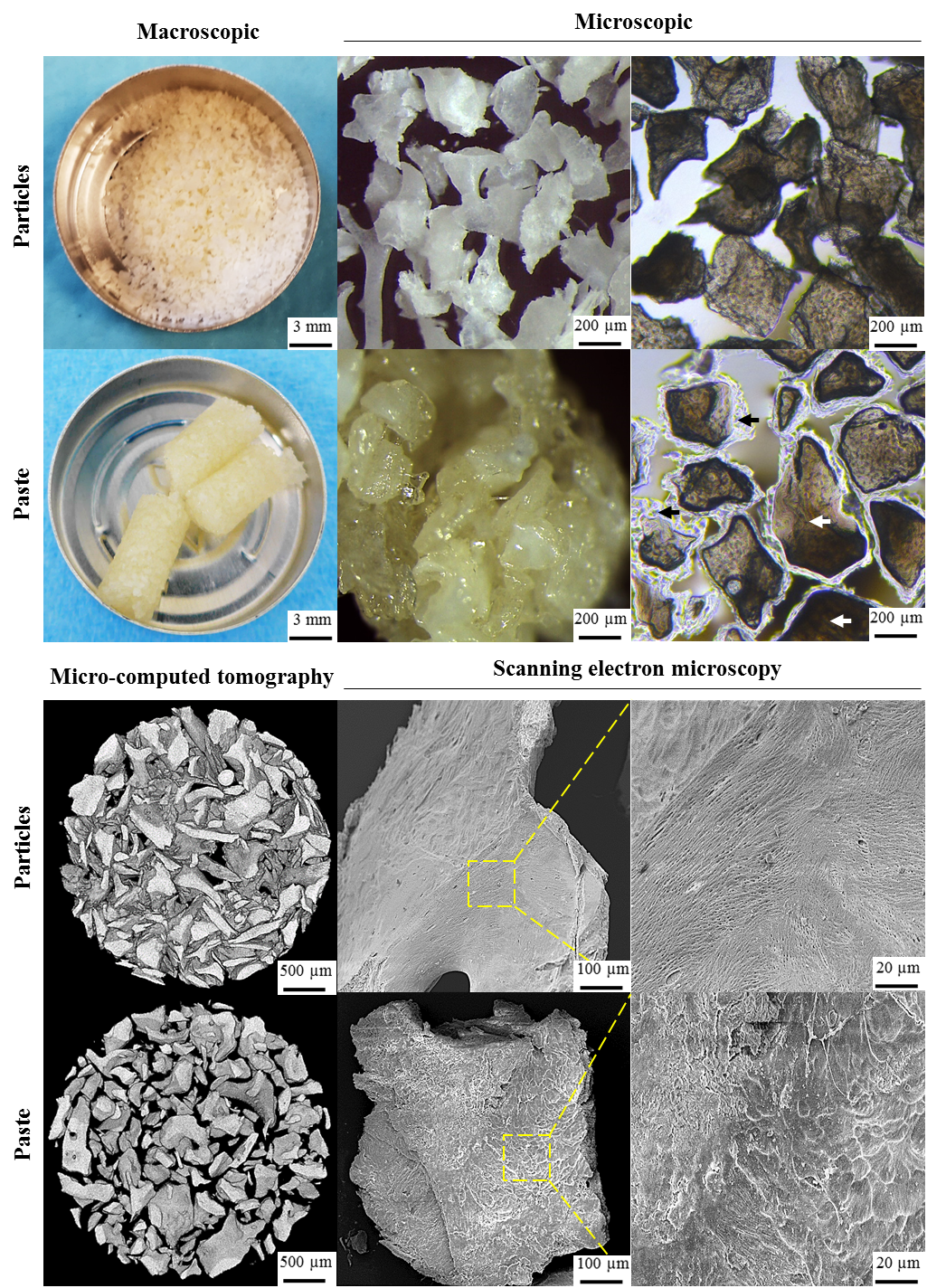


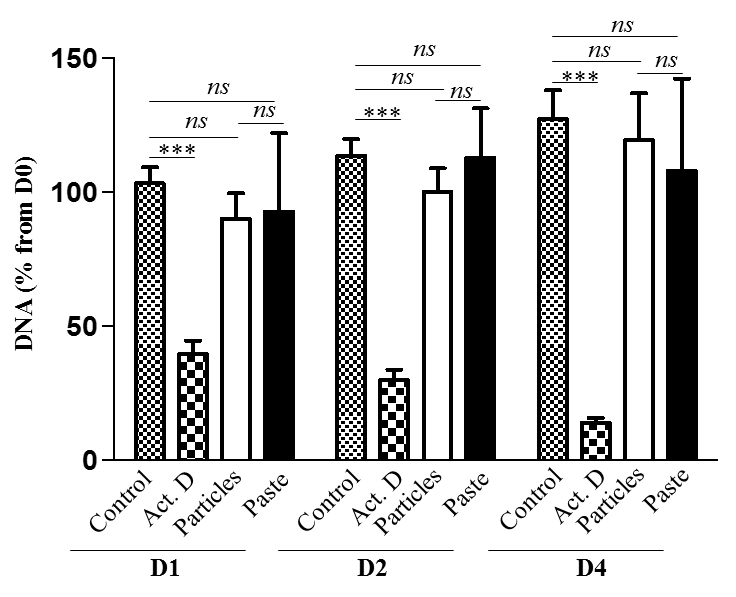


**Supplementary figure 1:** Images of the particular bone graft and the bone paste. The particular bone graft exhibited angular edges, while the bone paste was composed of smoother edges with a mineralized inner core (white arrows) and an outer demineralized bone matrix (black arrows). Micro-computed tomography revealed denser samples for the particular bone graft compared to the bone paste. Scanning electron microscopy images revealed the fibers of the bone matrix on the surface of the particular bone graft as well as the modified surface of the bone paste. The dashed yellow square indicates the magnified area.

**Supplementary figure 2:** Viability of two dimensional adherent hBM-MSCs cultured in contact with the particular bone graft or the bone paste or up to 4 days in 96-well plates. Quantification of the total DNA in hBM-MSCs in contact with the particular bone graft or the bone paste. The control represent the cells without the particular bone graft or the bone paste, Act. D represents the cells with 5 µg/mL of actinomycin D. All of the results are expressed as means ± SD (N = 3, n = 3; ***: *p* < 0.001).
